# An amplified sonodynamic therapy by a nanohybrid of titanium dioxide-gold-polyethylene glycol-curcumin: HeLa cancer cells treatment in 2D monolayer and 3D spheroid models

**DOI:** 10.1016/j.ultsonch.2023.106747

**Published:** 2023-12-25

**Authors:** H. Haghighi, N. Zahraie, M. Haghani, H. Heli, N. Sattarahmady

**Affiliations:** a Department of Medical Physics, School of Medicine, Shiraz University of Medical Sciences, Shiraz, Iran; b Nanomedicine and Nanobiology Research Center, Shiraz University of Medical Sciences, Shiraz, Iran; c Department of Radiology, School of Paramedical Science, Shiraz University of Medical Sciences, Shiraz, Iran

**Keywords:** Titania, Nanogold, Sonosensitizer, Sonotherapy, Wound healing

## Abstract

The utilization of ultrasound (US) to activate sonosensitizers for sonodynamic therapy (SDT) has faced challenges such as low activation efficiency and limited therapeutic outcomes, which have hampered its clinical applications. In this study, a nanohybrid of titanium dioxide-gold-polyethylene glycol-curcumin (TiO_2_-Au-PEG-Cur NH), as a novel US sensitizer, was synthesized, characterized, and applied for SDT of HeLa cancer cells in 2D monolayer model, and also a 3D spheroid model to bridge the gap between 2D cell culture and in vivo future studies. TiO_2_-Au-PEG-Cur NH contained TiO_2_ nanoparticles of 36 ± 11 nm in diameter, PEG-curcumin as a filler, and gold nanoparticles of 21 ± 7 nm in diameter with a high purity and a 35:17 of Ti:Au ratio (W/W), and it had a band gap of 2.4 eV, a zeta potential of –23 ± 7 mV, high stability upon US radiation cycles as well as one year storage. SDT of HeLa cells using TiO2-Au-PEG-Cur NH was investigated in the courses of cytotoxicity assessment in vitro, reactive oxygen species (ROS) generation capability, colony formation, cell migration, and the way to form spheroid. IC50 values of 122 and 38 μg mL^−1^ were obtained for TiO_2_-Au-PEG-Cur NH without and with US radiation, respectively. TiO_2_-Au-PEG-Cur NH not only exhibited an inherent capacity to generate ROS, but also represented an excellent therapeutic performance on the cancer cells through ROS generation and enhanced inhibitory effects on cell migration and spheroid formation.

## Highlights

• A nanohybrid of titanium dioxide-gold-polyethylene glycol-curcumin was synthesized and characterized.
• Sonodynamic therapy by the nanohybrid significantly killed the HeLa cells through synergism effects.
• The sonodynamic therapy rout was applicable in 2D monolayer and 3D spheroid models.
• The nanohybrid induced reactive oxygen specie generation upon ultrasound radiation.

## 1 Introduction

Cancer poses a severe threat to human health, with an estimated 1,958,310 new cases expected to be diagnosed in 2023, and a lethality rate of 31 % [1]. According to the International Agency for Research on Cancer, cervical cancer is regarded as a highly prevalent gynecological condition and is ranked as the fourth most common malignancy that endangers women's health [2]. Based on the American Health Rankings (2021), it has been reported that 66 % of patients diagnosed with cervical cancer have the potential to survive for at least five years [3]. Notwithstanding the ongoing progress in therapy, it is a prevalent occurrence that nearly 70 % of patients diagnosed with late-stage cervical cancer undergo metastasis. The main cause of this phenomenon is the inefficiency of surgical procedures in complete removal of malignant tissues, coupled with persistent resilience of stem cells in cervical cancer towards multiple therapeutic interventions [4].

Solid tumors can be treated in a number of methods including surgery, chemotherapy, and radiation therapy. Surgical treatments can result in bleeding, infection, and tumor recurrence as the most prevalent complications induced incomplete eradication of the entire tumor. The considerable toxicity of chemotherapy and the severe side effects of ionizing radiation treatment are only two of the numerous problems associated with these therapeutic modalities explored, which hinder their further clinical implementations [5], [6]. On the contrary, the application of novel therapeutic strategies, particularly those based on nanotechnology, can overcome the drawbacks of traditional medicines and offer a unique opportunity to improve the survival rate of cancer patients [5].

Alongside its significant role in clinical diagnostics, ultrasound (US) is also introduced as one of the most prominent noninvasive physical-irradiation source for cancer therapy with the advantages of being inexpensive, highly controllable, and easy-to-manufacture. High penetration ability is the most benefit of US radiation as it penetrates into cancer cells with minimal energy attenuation while sparing healthy tissues [7]. Low-intensity US (LIUS) and high-intensity focused US (HIFUS) are the two forms of US utilized in cancer treatment. The therapeutic efficacy of HIFUS treatment for deep-seated tumors is more limited compared to LIUS, making it ineffective in such cases due to the rapid energy attenuation along the ultrasonic channel [8], [9]. Sonodynamic therapy (SDT) that is a novel therapeutic approach and has been adopted from photodynamic therapy (PDT) employs a combination of LIUS and sonosensitizers for targeted eradication of cancer cells [10]. Although further research is required to completely find out the efficacy and potential adverse effects of SDT, it presents a potentially valuable noninvasive treatment option for cancer.

Ultrasonic waves have both the thermal and non-thermal effects on biological systems. Thermal effects occur as a result of US radiation absorption by targeted tissues, leading to a sudden increase in their temperature and subsequent alteration of the permeability of cell membranes. Non-thermal effects consist of a number of mechanisms, such as stable and transient cavitation, microstreaming and radiation forces, which can cause both temperature increases and mechanical tensions, such as microjets and microstreams [7]. The non-thermal interaction between US waves and tissues is responsible for the production of transient acoustic cavitation. This phenomenon takes place when the US pressure exceeds a certain threshold, resulting in the formation of gas-filled bubbles that undergo vigorous oscillation. Eventually, these bubbles collapse, give rise to intense shock waves, localize energy deposition, and elevate temperature [8]. The collapse of bubbles can be regarded as a small-scale sonochemical reactor that generates reactive oxygen species (ROS) through the decomposition of water molecules and induction of chemical transformations in the surrounding region (nearby region) [9]. Additionally, the production of sonoluminescence is one of the outcomes of cavitation [11]. Umemura et al. hypothesized that sonosensitizers could absorb the light emitted from bubbles in cavitation, thereby generating ROS via the same mechanism as photodynamic therapy [10].

Sonosensitizers are essential for SDT success and efficacy. However, accumulation under physiological conditions and elimination from circulation lead to formation of less ROS and ultimately decreased therapeutic efficacy [10], [12]. Nowadays, the emergence of nanotechnology has led to the development of nanoparticles (NPs)-based acoustic sensitizers [13], [14], [15], [16], [17], [18]. It has been demonstrated that the presence of NPs amplifies the cavitation effects and improves their efficiency by lowering the cavitation threshold [13], [14]. This activity of NPs is due to their abilities to stabilize nanobubbles (either at their surface or within their pores) and provide cavitation nucleation sites [19], [20].

Out of all NPs investigated as sonosensitizers, titanium dioxide NPs (TiO_2_NPs) possess several advantages that make them appropriate for the diagnosis and therapy of cancer. These NPs have properties such as biocompatibility, reusability, low toxicity, high thermal stability and large specific surface area, and photocatalytic, anti-microbial, anti-fungal, anti-inflammatory and anti-angiogenic activities. TiO_2_NPs can form nucleation centers that boost the formation of cavitation bubbles induced by US [21]. However, low quantum yield of US-triggered ROS generation by TiO_2_NPs is their major drawback arising from the rapid recombination of electron-hole pairs [22], [23]. Nonetheless, the integration of TiO_2_NPs with noble metal nanostructures such as gold, platinum or silver has demonstrated significant enhancements in ROS production under US stimulation, owing to the reduced band gap of noble metals-TiO_2_ nanomaterials [24]. This is because of the surface plasmon resonance (SPR) activity of noble metals that can enhance the US absorption of these nanostructures [24], [25], leading to prolonging the duration of electron acquisition in the excited state [24], [26]. Studies have also investigated the effectiveness of carbon doping with TiO_2_ to improve SDT treatment. The observed phenomenon was attributed to the high conductivity of carbon, which can increase the number of conduction of electrons and holes, thereby decreasing the band-gap of carbon-doped TiO_2_ [27].

Gold nanoparticles (AuNPs) have been explored as a potent acoustic sensor capable of amplifying both the mechanical and thermal interactions of US with tissues [28]. High biocompatibility, SPR activity, absorption by mammalian cells, photothermal activity and high atomic number enable AuNPs for great applications in biomedicine [15], [29], [30], [31], [32], [33]. As a result of facile surface modification, AuNPs can be easily absorbed by tumor cells and cleared from the body [34], [35]. Additionally, they would be useful as drug carriers because of their versatile surface chemistry [36]. Notably, a research indicated that AuNPs possessed special nucleation centers to accelerate the occurrence of inertial cavitation within biological systems [37].

The popularity of natural medicines has been increased due to their low toxicity, lack of adverse effects and affordability. As a naturally occurring compound, curcumin exhibits a wide range of anti-oxidant, anti-inflammatory, anti-carcinogenic, anti-diabetic, anti-parasitic, anti-bacterial, anti-fungal, anti-Alzheimer’s, immunity modulatory, wound healing and angiogenesis inhibitory properties [38], [39], [40]. In addition to its unique properties, curcumin also reduces tumor burden, induces death in cancer cells, and prevents cancer cells from multiplying [38], [39]. The anti-cancer effect of curcumin is characterized by its intricate involvement in multiple signaling pathways and interactions with various biomolecules. Curcumin impedes cell proliferation by blocking tumor cells in the G2/M phase, downregulates the PI3K-Akt-mTOR pathway by upregulating p53 and p21 genes, inhibiting β-catenin that attenuates the Wnt/β-catenin signaling pathway, and decreases the expression of GPC3 gene. Also, curcumin affects of cell cycle-related genes and inhibits tumor proliferation by controlling a number of miRNAs, which lower proteasome activity, prevents phosphorylation of JAK1, STAT1, and STAT3 proteins, and blocks mTOR and Notch1 pathways. Also, curcumin possesses the ability to modulate programmed cell death mechanisms, including autophagy, apoptosis, pyroptosis, paraptosis, and ferroptosis [41]. It can also represent sensitizing effect in PDT and SDT modalities [42], [43]. There is evidence that curcumin induces cell death in several types of cancer, including stomach and colon, human melanoma, and lung without having significant cytotoxic effects on healthy cells [44]. Regardless of its potential therapeutic benefits, the lack of bioavailability, poor solubility, low absorption, rapid metabolism, high excretion rate, limited tissue distribution, and poor stability of the curcumin present significant challenges [45]. Nanoformulations of curcumin such as encapsulating in or binding with nanocarriers, or conjugating with PEG can solve the curcumin insolubility, increase its circulation time, and may also mitigate its disadvantages [40], [46]. Encapsulating or binding with metal oxides such as TiO_2_, ZnO, Fe_3_O_4_, CeO_2_ and CuO protects curcumin from hydrolysis and phagocytosis, leading to its improved aqueous contact, blood circulation, stability, and bioavailability [47]. PEG conjugation prevents nanoparticles from absorbing by opsonin proteins, allowing them to circulate longer and reach their targets [48].

In the present study, a nanohybrid titanium dioxide-gold-polyethylene glycol-curcumin (TiO_2_-Au-PEG-Cur NH) was synthesized and physicochemically characterized, and its treatment efficiency to kill monolayers as well as 3D spheroids in gel agarose phantoms of HeLa cancer cells was evaluated under US radiation. TiO_2_-Au-PEG-Cur NH potential to impede cell migration and colony formation was also examined. The ability of TiO_2_-Au-PEG-Cur NH to generate ROS upon US radiation was also inspected.

## 2 Materials and methods

### 2.1 Materials

All chemicals were purchased from Sigma Chemicals Co. (USA), Scharlau Chemie Co. (Spain), or Merck Co. (Germany). The chemicals were used without further purification. Prior to usage, all glassware was cleaned with newly prepared aqua regia, followed by rinsing with deionized (DI) water. DI water was used for final washing and solutions preparation throughout the study.

### 2.2 Synthesis of TiO_2_-Au-PEG-Cur NH

A schematic presentation of the synthesis procedure is shown in supplementary material S1. TiO_2_NPs were firstly synthesized based on the method reported previously [29] by some modifications. 6 mL of TiCl_4_ was dropped into 80 mL of stirred DI water with continuous stirring for 10 min. The solvent of the obtained suspension was evaporated at ∼ 90 °C. The obtained powder was finally heated at 450 °C for 60 min to obtain TiO_2_NPs. Then, 50 mg of the prepared TiO_2_NPs was completely dispersed in 50 mL of DI water by a horn-sonicator. Afterward, 10 mL solution containing 34 mg HAuCl_4_ dissolved in DI water was added to the TiO_2_ dispersion. Subsequently, 4.0 mg curcumin was mixed with 20 mL of PEG 600 and dropwise added to the above mixture. Following 15 min, 45 mL solution containing 17 mg NaBH_4_ dissolved in cold DI water was drop-wise added to the mixture, and stirring was continued for 2 h. Finally, the resulting product was centrifuged at 5000 rpm for 30 min to obtain TiO_2_-Au-PEG-Cur NH.

### 2.3 Characterization of TiO_2_-Au-PEG-Cur NH

Morphology and size of TiO_2_-Au-PEG-Cur NH were characterized using a Zeiss Sigma-IGMA/VP field emission scanning electron microscope (FESEM, Germany) with energy-dispersive X-ray spectroscopy (EDS) and elemental mapping capabilities. The zeta potential of TiO_2_-Au-PEG-Cur NH was measured using a SZ-100 HORIBA instrument (Japan). UV–vis absorption spectra of TiO_2_-Au-PEG-Cur NH suspensions were measured using a Rayleigh UV-2601 spectrophotometer (China). The spectra were recorded in a range of 200 to 900 nm. To calculate the band gap energy of TiO_2_-Au-PEG-Cur NH, we employed Tauc’s method [49].

### 2.4 Stability assessment of TiO_2_-Au-PEG-Cur NH

Thermal stability of TiO_2_-Au-PEG-Cur NH under US radiation was examined by subjecting a 50 µg mL^−1^ of its dispersion in DI water to five cycles of exposure at a power density of 1.0 W cm^−2^, a frequency of 1.0 MHz, a duty cycle of 10 % (consisting on/off time of 1/9 ms), and recording temperature variations every one minute using a thermoprobe of Lutron (Taiwan) with an accuracy of 0.01 °C. After reaching temperature to a plateau, the US device (vide infra, section 2.5) was switched off, and the solution container was left until its temperature approximately returned to the initial value. In addition, UV–vis absorption spectra of the as-synthesized TiO_2_-Au-PEG-Cur NH after one year storage in 0.1 mol/L phosphate-buffered saline (PBS) were recorded.

### 2.5 US instrument and exposure set up

HeLa human cervical cancer cells (vide infra, section 2.6) were plated in separated wells and the center of a gel-coated unfocused US planar transducer of Novin (Iran) was positioned beneath the middle of each well. The US radiation parameters were frequency of 1.0 MHz, output power density of 1.0 W cm^−2^ and duty cycle of 10 % for 10 min. All intensity values were reported as the spatial average-temporal average (I_SATA_).

### 2.6 In vitro cytotoxicity assay

HeLa human cervical cancer cells were acquired from Pasteur Institute (Iran) and cultured in the standard Dulbecco's Modified Eagle Medium (DMEM) of Gibco (USA) supplemented with 10 % FBS and 1 % penicillin/streptomycin at 37 °C in a humidified atmosphere with 5 % CO_2_. HeLa cells at 1 × 10^4^ cells/well in the logarithmic growth phase were seeded in 96-well cell culture plates to assess the cytotoxicity of the cells treated with TiO_2_-Au-PEG-Cur NH, US radiation, or combination of both treatments in the following groups:

­ US-/NH–: cells without any treatment (control).
­ US-/NH+: cells treated with different concentrations of TiO_2_-Au-PEG-Cur NH of 10, 25, 50, and 100 μg mL^−1^ (no US radiation).
­ US+1/NH–: cells treated with US radiation (no incubation with TiO_2_-Au-PEG-Cur NH).
­ US+2/NH–: cells treated with US radiation two times with one hour interval (no incubation with TiO_2_-Au-PEG-Cur NH).
­ US+1/NH+: cells treated firstly with different concentrations of TiO_2_-Au-PEG-Cur NH of 10, 25, 50, and 100 μg mL^−1^ and after one hour, with US radiation
­ US+2/NH+: cells treated firstly with different concentrations of TiO_2_-Au-PEG-Cur NH of 10, 25, 50, and 100 μg mL^−1^ and after one hour, with US radiation two times with one hour interval.

After treatment, all the groups were subsequently incubated overnight at 37 °C in a humidified atmosphere with 5 % CO_2_. The cell groups were prepared at least in triplicate. The relative cell viabilities were measured using a standard colorimetric MTT (3-[4,5-dimethylthiazol-2-yl]-2,5 diphenyl tetrazolium bromide) assay. Cell viabilities were reported as the ratio of optical densities at 570 nm of experimental groups and the control using a microplate reader of Biotek (USA).

### 2.7 ROS generation assays

#### 2.7.1 Cell-free ROS production ability of TiO_2_-Au-PEG-Cur NH

To evaluate the inherent capacity of TiO_2_-Au-PEG-Cur NH to trigger ROS production, we used H_2_DCF (2′,7′-dichlorodihydrofluorescein) without cellular reactions. H_2_DCF was prepared by deacetylation of H_2_DCF-DA (2′,7′-dichlorodihydrofluorescein diacetate). H_2_DCF was incubated with two concentrations of TiO_2_-Au-PEG-Cur NH of 10 and 100 μg mL^−1^ to assess TiO_2_-Au-PEG-Cur NH intrinsic capacity to convert H_2_DCF into highly fluorescent DCF (2′,7′-dichlorofluorescein).

Firstly, a 1.0 mmol/L stock solution of H_2_DCF-DA dissolved in methanol was mixed with 40 mL of 100 mmol/L NaOH and stirred for 30 min at room temperature in a light-resistant beaker. 150 mL of a 33 mmol/L NaH_2_PO_4_ solution was eventually added to obtain a 50 µmol/L H_2_DCF solution [50]. Then, 250 μL of 50 µmol/L H_2_DCF was mixed with 250 μL of TiO_2_-Au-PEG-Cur NH (10 and 100 μg mL^−1^) in a fluorescence cuvette, and US radiation was performed at a frequency of 1.0 MHz, an output power density of 1.0 W cm^−2^, and duty cycle of 10 % for 10 min. After one hour incubation at room temperature in dark, US radiation was repeated. Fluorescence intensities were then measured for samples after both the first and second times of US radiation, using a spectrofluorometer of Varian (USA). PBS was considered as a control.

#### 2.7.2 Intracellular ROS production ability of TiO_2_-Au-PEG-Cur NH

To evaluate the capability of TiO_2_-Au-PEG-Cur NH to trigger intracellular ROS production, we used H_2_DCF-DA. H_2_DCF-DA permeates the cells, is transformed into a non-fluorescent derivative by cellular esterase, and is oxidized by intracellular ROS to create DCF. For this assay, the cells were incubated with H_2_DCF-DA of 50 µmol/L for 30 min. Then, US radiation was performed. After that, the cells were rinsed with PBS to remove the extracellular DCF and lysed with a lysis buffer. The intensity of fluorescence emission was measured at 485 nm excitation and 520 nm emission wavelengths to quantify the ROS production level in each cell groups (vide supra, section 2.6), using a microplate reader of Biotek (USA).

### 2.8 Clonogenic assay

The colony formation assay was used to evaluate the capacity of solitary cells to form colonies [51]. HeLa cells at 1 × 10^3^ cells/well in the logarithmic growth phase were seeded in 6-well cell culture plates to assess the clonogenic assay of the cells treated with TiO_2_-Au-PEG-Cur NH, US radiation, or combination of both treatments in the following groups:

- US-/NH–: cells without any treatment (control).
- US-/NH+: cells treated with TiO_2_-Au-PEG-Cur NH of 50 μg mL^−1^ (no US radiation).
- US+1/NH–: cells treated with US radiation (no incubation with TiO_2_-Au-PEG-Cur NH).
- US+2/NH–: cells treated with US radiation two times with one hour interval (no incubation with TiO_2_-Au-PEG-Cur NH).
- US+1/NH+: cells treated firstly with TiO_2_-Au-PEG-Cur NH of 50 μg mL^−1^ and after one hour, with US radiation
- US+2/NH+: cells treated firstly with TiO_2_-Au-PEG-Cur NH of 50 μg mL^−1^ and after one hour, with US radiation two times with one hour interval.

After treatment, all the groups were subsequently incubated for two weeks at 37 °C in a humidified atmosphere with 5 % CO_2_. The culture medium was replaced twice per week. After two weeks of incubation, the cells were rinsed with PBS and fixed with cold methanol for 10 min. A solution of crystal violet of 0.5 % was used to stain the cells to identify formation and enumerate colonies containing at least fifty cells. Then, the plating efficiency (PE) and cell survival fraction (SF) were calculated according to:

(1) PE%=numberofcountedcolonies/numberofseededcells

(2) SF%=numberofcountedcoloniesaftertreatment/(numberofseededcells×PE)

The cell groups were prepared in triplicate.

### 2.9 Wound healing assay

To assess migration and metastatic potentials of HeLa cell monolayers, we seeded the cells at 7 × 10^4^ cells/well in the logarithmic growth phase in 24-well cell culture plates and incubated at 37 °C in a humidified atmosphere with 5 % CO_2_ to attain 80 % cell confluence. Then, a manual scratch along the center of each well was created by a 100-μL pipette tip, and optical images were recorded from the plates. After that, the cells were treated with TiO_2_-Au-PEG-Cur NH, US radiation, or combination of both treatments in the cell groups similar as mentioned in section 2.9. Optical images were then recorded in a time-lapse manner (after 8, 24, and 48 h of treatment) to inspect the cells migration into the wound. Wound areas were measured by ImageJ program, and migration ability was expressed as the percentage of wound closure calculated as:

(3) $$\mathrm{Wound}\;\mathrm{closure}\%=\left(\mathrm{initial}\;\mathrm{scratch}\;\mathrm{area}-final\;scratch\;area\right)/initial\;scratch\;area$$

### 2.10 Spheroid formation

Into wells of a 96-well plate, 50 μL of an autoclaved 2 % agarose solution was added followed by addition of HeLa cells at 1 × 10^4^ cells/well. Images were recorded at 24, 48, 72, and 96 h after cell seeding (under incubation conditions) to follow the spheroid formation. After 96 h of incubation, the cells medium was treated with TiO_2_-Au-PEG-Cur NH of 50 μg mL^−1^, US radiation, or combination of both treatments in the following groups:

- US-/NH–: cells without any treatment (control).
- US-/NH+: cells treated with TiO_2_-Au-PEG-Cur NH of 50 μg mL^−1^ (no US radiation).
- US+2/NH–: cells treated with US radiation two times with one hour interval (no incubation with TiO_2_-Au-PEG-Cur NH).
- US+2/NH+: cells treated firstly with TiO_2_-Au-PEG-Cur NH of 50 μg mL^−1^ and after one hour, with US radiation two times with one hour interval.

After treatment, all the groups were subsequently incubated overnight at 37 °C in a humidified atmosphere with 5 % CO_2_. The cell groups were prepared in triplicate. Relative spheroid viabilities were evaluated using a resazurin viability assay by measuring the intensity of resazurin fluorescence at 544 nm excitation and 600 nm emission wavelengths using a microplate reader of Biotek (USA).

### 2.11 Evaluation of domination of synergism effects

For evaluation of the domination of synergism effects between US radiation and TiO_2_-Au-PEG-Cur NH, as two factors during the HeLa cell SDT, combination indices (CIs) were calculated according to:

(4) CI = V_US_ × V_NH_ / V_US/NH_

where V_US_, V_NH_ and V_US/NH_ are the effects of US radiation, TiO_2_-Au-PEG-Cur NH and their combination, respectively. For CI > 1, CI = 1 and CI < 1, synergism, additive and antagonism effects will be dominated, respectively.

### 2.12 Statistical analysis

Data obtained from the experiments were reported as mean and standard deviation, and a minimum of three measurements were conducted to obtain the results. The statistical significance of the values was evaluated by GraphPad Prism 9 software, employing Two-tailed Student's *t*-test and non-parametric test of Kruskal-Wallis. Statistical significance was considered as p-values less than 0.05.

## 3 Results and discussion

### 3.1 Characterization of TiO_2_-Au-PEG-Cur NH

**Fig. 1:**
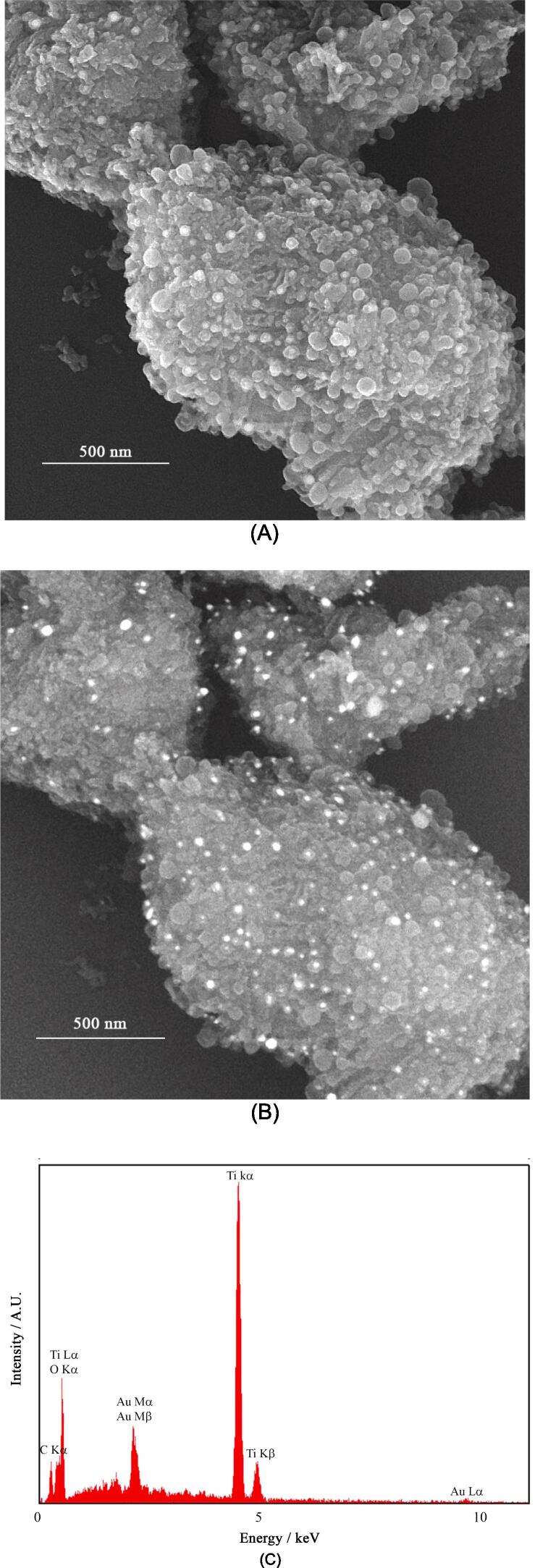
FESEM images of TiO_2_-Au-PEG-Cur NH recorded by the secondary electron (A) and back scattered electron (B) detectors, and the corresponding EDS (C).

Size, shape, and surface chemistry of nanomaterials with application in cancer therapy play crucial roles in their behavior in vivo and ultimately determine their anti-tumor effects [52], [53]. FESEM images of TiO_2_-Au-PEG-Cur NH recorded by both secondary- and back scattered-electron detectors are presented in Fig. 1A and B. The images indicated that TiO_2_-Au-PEG-Cur NH comprised TiO_2_NPs with a mean diameter of 36 ± 11 nm (n = 100), PEG-curcumin as a TiO_2_NPs filler, and AuNPs adhered to these with a mean diameter of 21 ± 7 nm (n = 100). Blood vessels in normal tissues have a pore size of < 12 nm, a previous research suggested that nanoparticles with a diameter of > 12 nm selectively localize in malignancies [54], and a mathematical model showed that particles less than 60 nm were able to penetrate the endothelial barrier more easily because of reduced interactions [55]. Hence, a size range of 12 to 60 nm is favorable for a nanoparticle to effectively penetrate and internalize tumor tissues. Therefore, TiO_2_-Au-PEG-Cur NH would effectively internalize and penetrate into tumor tissues in terms of size. In addition, its spherical shape can contribute to biocompatibility and stability. Elemental mapping images presented in supplementary material S2 also indicated that all the components of TiO_2_-Au-PEG-Cur NH were uniformly distributed within together and formed a homogenous composite. EDS results that are presented in Fig. 1C indicated the presence all of the components of TiO_2_-Au-PEG-Cur NH with relative weight ratio of 35:17 for Ti:Au.

**Fig. 2:**
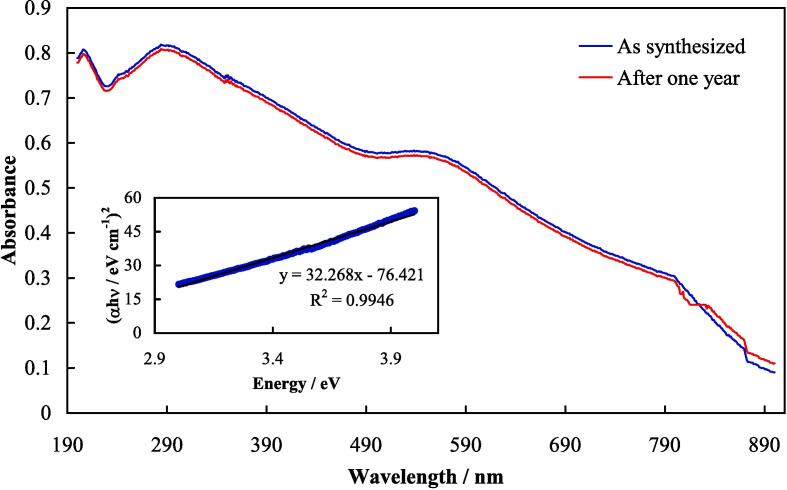
Typical UV–vis absorption spectra of as synthesized TiO_2_-Au-PEG-Cur NH and after one year storage. Inset: A Tauc plot derived from the UV–vis spectrum of TiO_2_-Au-PEG-Cur NH.

A typical UV–vis absorption spectrum of TiO_2_-Au-PEG-Cur NH is shown in Fig. 2, representing absorption peaks at about 300 and 540 nm attributed to TiO_2_NPs absorption and AuNPs SPR, respectively. By using UV–vis spectra of a semiconductor, its band gap can be determined by Tauc plot based on the equation [49]:

(5) (αhʋ)^1/γ^ = C (hʋ - E_g_)

where, α, h, ʋ and E_g_ are optical absorption coefficient, Planck’s constant, photon’s frequency and band gap energy, respectively, and C is a constant. Also, γ is a factor which depends on the nature of the electron transition and is equal to 1/2 or 2 for the direct and indirect transition band gaps, respectively. α, optical absorbance (A) and light pass length (d) are related to each other by the equation [49]:

(6) α = (2.303A) / d

Photon energy (hʋ) is also related to wavelength (λ) by the equation [49]:

(7) hʋ = 1240 / λ

Tauc plot derived from the UV–vis spectrum of TiO_2_-Au-PEG-Cur NH is presented in Fig. 2, inset, revealing a band gap of 2.4 eV. It was much narrower than that for pure TiO_2_ (3.20 eV [24]). This narrower band gap leads to prevention of fast recombination of excited electrons and holes, thereby enhancing the quantum yield of ROS generation [24].

Zeta potential of TiO_2_-Au-PEG-Cur NH was measured to be –23 ± 7 mV (supplementary material S3). The value of this zeta potential can guarantee the TiO_2_-Au-PEG-Cur NH resistance toward particle aggregation, and its sign leads to decrement in the adsorption by the serum proteins [56].

Time stability of TiO_2_-Au-PEG-Cur NH upon storage in PBS at 4 °C was evaluated by recording UV–vis spectra for the synthesis sample as well as after one year storage; the spectra are depicted in Fig. 2. Change in the spectra was negligible, indicating at least one year of stability for TiO_2_-Au-PEG-Cur NH. On the other side, thermal stability of TiO_2_-Au-PEG-Cur NH upon five cycles of heating by US radiation (section 2.5)/cooling was inspected, and the results are represented in supplementary material S4. The heating/cooling data indicated that TiO_2_-Au-PEG-Cur NH was stable when used as a sonosensitizer.

### 3.2 In vitro cytotoxicity assessment

**Fig. 3:**
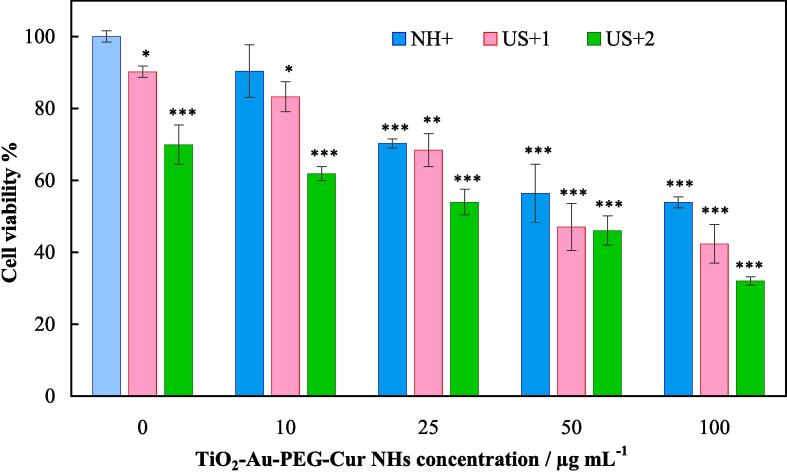
In vitro viability percents of HeLa cells treated in different groups. * indicates significant differences (P-value < 0.05). ** indicates very significant differences (P-value < 0.01).

The biocompatibility of TiO_2_-Au-PEG-Cur NH toward Hela cells was assessed using the MTT assay (supplementary material S5), and the results are shown in Fig. 3 representing no significant cytotoxicity up to the concentration of 50 μg mL^−1^, and a high IC50 value of 122 μg mL^−1^ was estimated. A comparison of IC50 values reported for some potential sonosensitizers is presented in supplementary material S6, indicating a high level of biocompatibility of TiO_2_-Au-PEG-Cur NH.

SDT of Hela cells using TiO_2_-Au-PEG-Cur NH was evaluated by the MTT assay, and the results are also shown in Fig. 3. The results indicated that US radiation of TiO_2_-Au-PEG-Cur NH induced cell killing effects, and higher killing effects were observed upon increment in the US radiation dose, increment in the TiO_2_-Au-PEG-Cur NH concentration significantly reduced the cell survival, and the highest killing effect was attained at the highest TiO_2_-Au-PEG-Cur NH concentration and higher US radiation dose (TiO_2_-Au-PEG-Cur NH of 100 μg mL^−1^ and US radiation two times with one hour interval). Using the data, an IC50 value of 38 μg mL^−1^ was also obtained for US+2/NH+ group. Calculated CIs for the treatment groups revealed domination of additive effects in US+1/NH + and US+2/NH + groups at TiO_2_-Au-PEG-Cur NH concentrations of 10 and 25 µg mL^−1^ (CIs of 1.0 and 0.97, respectively), and synergism effects in those groups at TiO_2_-Au-PEG-Cur NH concentrations of 50 and 100 µg mL^−1^ (CIs of 1.2 and 1.2, respectively). Therefore, a combined application of TiO_2_-Au-PEG-Cur NH and US radiation is introduced as an efficient method for eliminating cancer cells with ascendancy of synergistic effects. A comparison between sonosensitizers comprised the components of TiO_2_-Au-PEG-Cur NH is presented in supplementary material S7.

### 3.3 Evaluation of ROS generation

**Fig. 4:**
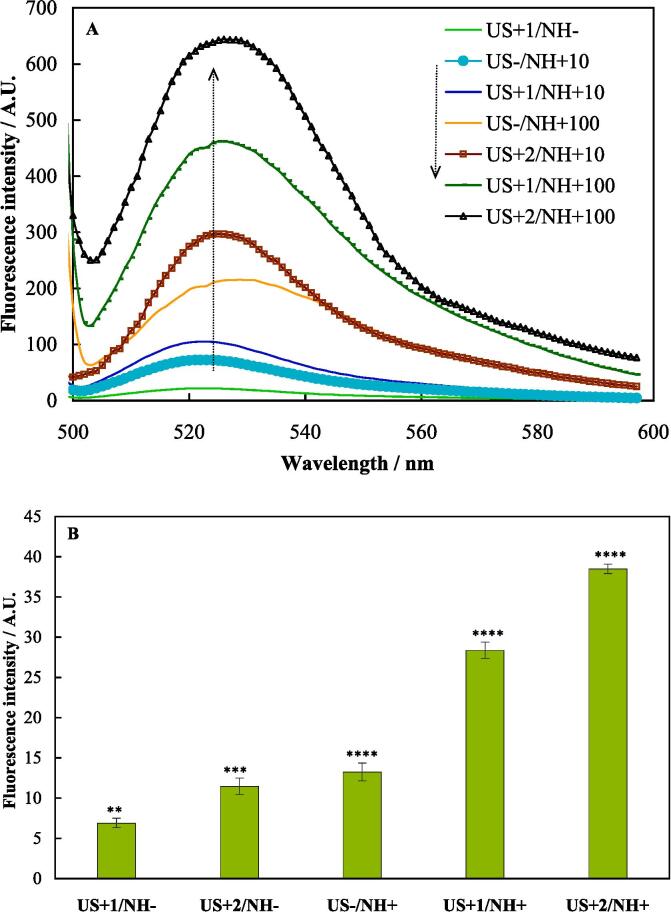
Background-corrected fluorescence emission spectra of DCF upon US radiation, incubation with two concentrations of TiO_2_-Au-PEG-Cur NH of 10 and 100 μg mL^−1^ or both of a H_2_DCF solution (A). Fluorescence intensity of intracellular DCF in different cell groups (B). * indicates significant differences (P-value < 0.05). *** indicates high significant differences (P-value < 0.001). **** indicates extremely significant differences (P-value < 0.0001).

The inherent capacity of TiO_2_-Au-PEG-Cur NH to generate ROS was evaluated by recording fluorescence emission of DCF (Fig. 4A). The results indicated that US radiation of PBS led to generation of a bit ROS, and TiO_2_-Au-PEG-Cur NH had a potential to generate a considerable level of ROS (higher generated ROS at higher concentration) that was accelerated by US radiation. Moreover, the findings indicated that the quantity of ROS produced by TiO_2_-Au-PEG-Cur NH escalated with repeated US radiation. The results confirmed the sonosensitizing activity of TiO_2_-Au-PEG-Cur NH to be potent.

The capability of TiO_2_-Au-PEG-Cur NH to generate intracellular ROS was evaluated by recording fluorescence emission of intracellular DCF (Fig. 4B). The results indicated that US radiation alone of the cells effectively generated intracellular ROS, TiO_2_-Au-PEG-Cur NH affection of the cells more effectively generated intracellular ROS, and SDT of the cells using TiO_2_-Au-PEG-Cur NH generated the most intracellular ROS. The results were in accordance with the results of in vitro cytotoxicity assessment. Since the excellent electron conductivity of AuNPs effectively inhibits the recombination of electron-hole pairs [24] and AuNPs are capable of capturing and transferring the excited electrons, integration of AuNPs with TiO_2_NPs leads to efficient separation and prolonged lifetime of electron-hole, and significantly enhances the sonocatalytic performance of TiO_2_NPs and generation of a larger quantity of ROS (such as singlet oxygen (^1^O_2_), superoxide radical (O_2_^−^) and hydroxyl radical (•OH)) with potent oxidation capabilities (supplementary material S8). In contact with cells, ROS damages proteins, lipids, and DNA within the cells, and makes protein inactivation, protein cross-linking, and DNA strand breakage.

### 3.4 Influence of SDT on colony formation

**Fig. 5:**
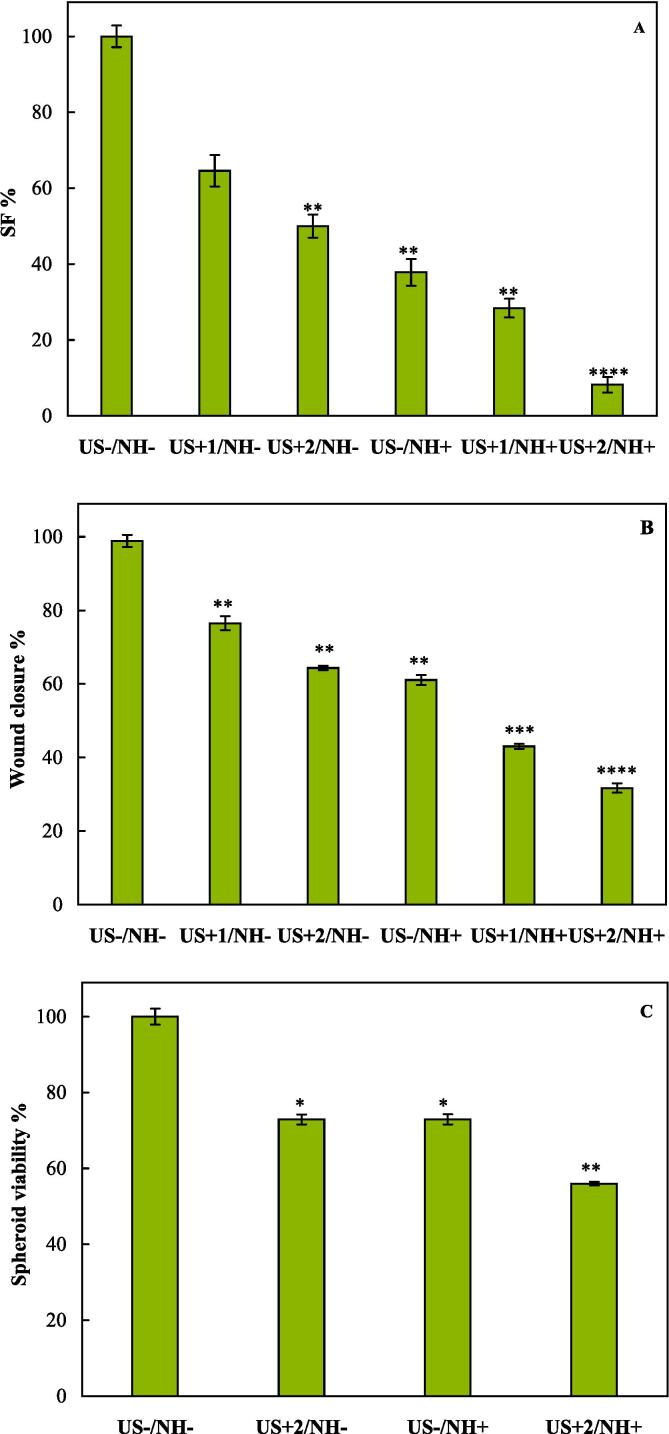
SF percents of HeLa cells for different treatment groups (A). Wound closure percents of HeLa cells for different treatment groups (B). Spheroid viability percents of HeLa cells treated in different groups (C). * indicates significant differences (P-value < 0.05). ** indicates very significant differences (P-value < 0.01). *** indicates high significant differences (P-value < 0.001).

Since tumors recurrence after treatment depends on the ability of surviving cells to proliferate, clonogenic capacity of HeLa cells after SDT was evaluated. Photographs were recorded from colony formation of HeLa cells for different treatment groups (as presented in supplementary material S9), and the results of clonogenic survival assay for SDT of HeLa cells are presented in Fig. 5A. The results in agreement with those obtained for ROS generation indicated that cells treated with either with US radiation or TiO_2_-Au-PEG-Cur NH, or both were inhibited to form colony in a manner of US-/NH–<US+1/NH–<US+2/NH–<US-/NH+<US+1/NH+<US+2/NH+. Calculated CIs for colony formation inhibition also showed that HeLa cells treatment in all the groups represented additive effect between US radiation and TiO_2_-Au-PEG-Cur NH, while in the US+2/NH+ group these two parameters affected the cells in a synergistic manner and resulted in a SF of ∼ 8 %. Therefore, combination of TiO_2_-Au-PEG-Cur NH and US radiation is more efficient than either US radiation or TiO_2_-Au-PEG-Cur NH alone to reduce the colony numbers of HeLa cells. Thus, SDT inhibits the ability of treatment-surviving cells to form colonies that is in line with the MTT assay results.

### 3.5 Influence of SDT on cell migration

The ability to heal a wound is based on cell migration into the wound area. A wound healing assay was performed to investigate whether SDT by TiO_2_-Au-PEG-Cur NH influenced the migration ability of HeLa cells. Photographs were recorded from the cells at different time intervals, as presented in supplementary material S10, and the percentage of wound closure in different groups is shown in Fig. 5B. 24 h after treatment, cells in the US+1/NH + and US+2/NH+ groups showed minimal movement into the interspace of the wound, while in others the cells gradually grew into the wound gap. 48 h after treatment, cells in the US+1/NH+ and US+2/NH+ groups had not yet fully proliferated in the wound area, resulting in a significantly lower cell density, compared to the US-/NH– group in a manner that the mean wound closures of US+1/NH+ and US+2/NH+ groups decreased by almost 57 % and 69 %, respectively. Therefore, the synergetic effect of TiO_2_-Au-PEG-Cur NH and US radiation significantly enhanced the cell migration inhibition.

### 3.6 Spheroid formation assessment

Compared to 2D cell culture, assessment of cell spheroids in 3D platforms has the advantages of ability to replicate the complex cell–cell and cell-matrix interactions and diverse diffusion/transport conditions providing an accurate representation of nanomaterials toxicity within the body. Sonosensitizer accumulation in the spheroids is influenced by multiple factors, and the presence of hypoxia and altered metabolism can affect the cyototoxic responses. Furthermore, the slower proliferation of spheroids, compared to 2D cell culture, contributes to lower cytotoxicity. In 2D culture, the cells experience uniform oxygen levels, whereas spheroids reduce oxygen diffusion, leading to reduced ROS production and lower efficacy of SDT in the core [57]. A timeline for determination of spheroid formation is depicted in supplementary material S11, and the results are summarized in Fig. 5C. Based on the results, iterative utilization of US radiation without TiO_2_-Au-PEG-Cur NH (US+2/NH– group) and also cells treated with TiO_2_-Au-PEG-Cur NH of 50 μg mL^−1^ without US radiation (US-/NH+ group) caused 27 % reduction in the viability. The results of the viability assay indicated that TiO_2_-Au-PEG-Cur NH efficacy in response to US radiation treatment exhibited increased therapeutic efficacy (44 % reduction in the viability in US+2/NH+ group) due to their higher cavitation activity.

Domination of hypoxia, alteration in metabolism and slow downing proliferation of cells in spheroids induce lower cytotoxic response, compared to 2D cell culture. Although assessment of toxicity behavior of a sonosensitizer and ultrasound radiation using in vitro 2D cell culture models offer valuable and significant information, they cannot accurately forecast in vivo toxicity and other biological effects. In 2D culture, cells experience uniform oxygen levels, whereas spheroids have reduced oxygen diffusion, leading to reduced ROS. The existence of the extracellular matrix in 3D cultures induces restriction in the movement and even distribution of substances and molecules [58], [59]. The presence of diffusion limitations and low oxygen levels in spheroids are anticipated to induce substantial alterations in cellular metabolic pathways.

## 4 Conclusion

A novel nanosonosensitizer containing sonoabsorbing materials (AuNPs and TiO_2_NPs), an electron-hole recombination inhibitor and band gap narrower of TiO_2_NPs (AuNPs), a naturally occurring anticancer agent (curcumin), and a biocompatible polymer linker (PEG) for SDT of cervix cancer cells and their spheroids was introduced. US radiation led to > 3 times decrement in IC50 of TiO_2_-Au-PEG-Cur NH in a manner that a remarkable effectiveness in destruction of the cancer cells, suppressing cellular migration and colon formation were attained. TiO_2_-Au-PEG-Cur NH promoted formation of cavitation bubbles as well as ROS generation on its activated sonocatalytic sites. Replacement of curcumin with its other derivatives or anticancer drugs, co-loading of anticancer drugs with curcumin, application of other shapes of gold nanostructures, PDT or SDT-co-PDT of cancer cells using TiO_2_-Au-PEG-Cur NH, SDT of other cancerous cells, and development of SDT using TiO_2_-Au-PEG-Cur NH in animal models could be the future of studies based on the presented research.

## Conflict of interest

There was no conflict of interest.

## Data availability statement

The data related to this research was included in the manuscript files.

## CRediT authorship contribution statement

**H. Haghighi:** Formal analysis, Investigation, Validation, Writing – review & editing. **N. Zahraie:** Data curation, Visualization. **M. Haghani:** Data curation, Methodology. **H. Heli:** Formal analysis, Investigation, Validation, Writing – review & editing. **N. Sattarahmady:** Conceptualization, Funding acquisition, Investigation, Project administration, Resources, Supervision, Validation, Writing – review & editing.

## Declaration of competing interest

The authors declare that they have no known competing financial interests or personal relationships that could have appeared to influence the work reported in this paper.
