# Supplementary material for: An amplified sonodynamic therapy by a nanohybrid of titanium dioxide-gold-polyethylene glycol-curcumin: HeLa cancer cells treatment in 2D monolayer and 3D spheroid models
Source: Ultrason Sonochem. 2023 Dec 25;102:106747. doi: 10.1016/j.ultsonch.2023.106747 (PMC10765485; doi:10.1016/j.ultsonch.2023.106747)
Supplement: Supplementary data 1 [file mmc1.pdf]

Legends for supplementary materials:

S1: A schematic presentation of the synthesis procedure of TiO_2-Au-PEG-Cur NH.

S2: Elemental mapping images TiO_2-Au-PEG-Cur NH.

S3: Zeta potential of TiO_2-Au-PEG-Cur NH.

S4: Temperature changes of a dispersion of TiO_2-Au-PEG-Cur NH of 50 $\mu g\ mL^{-1}$ upon US radiation for five cycles of heating/cooling.

S5: MTT assay set up assess the TiO_2-Au-PEG-Cur NH cytotoxicity.

S6: A comparison of IC50 values reported for some potential sonosensitizers.

S7: A comparison between sonosensitizers comprised the components of TiO_2-Au-PEG-Cur NH.

S8: Schematic illustration of SDT using TiO_2-Au-PEG-Cur NH through ROS generation.

S9: Photographs recorded from colony formation of HeLa cells for different treatment groups.

S10: Photographs recorded from HeLa cells at different time intervals to evaluate migration ability.

S11: A timeline photographs recorded from HeLa cells during spheroid formation.
