# Supplementary material for: An amplified sonodynamic therapy by a nanohybrid of titanium dioxide-gold-polyethylene glycol-curcumin: HeLa cancer cells treatment in 2D monolayer and 3D spheroid models
Source: Ultrason Sonochem. 2023 Dec 25;102:106747. doi: 10.1016/j.ultsonch.2023.106747 (PMC10765485; doi:10.1016/j.ultsonch.2023.106747)
Supplement: Supplementary data 2 [file mmc2.pdf]

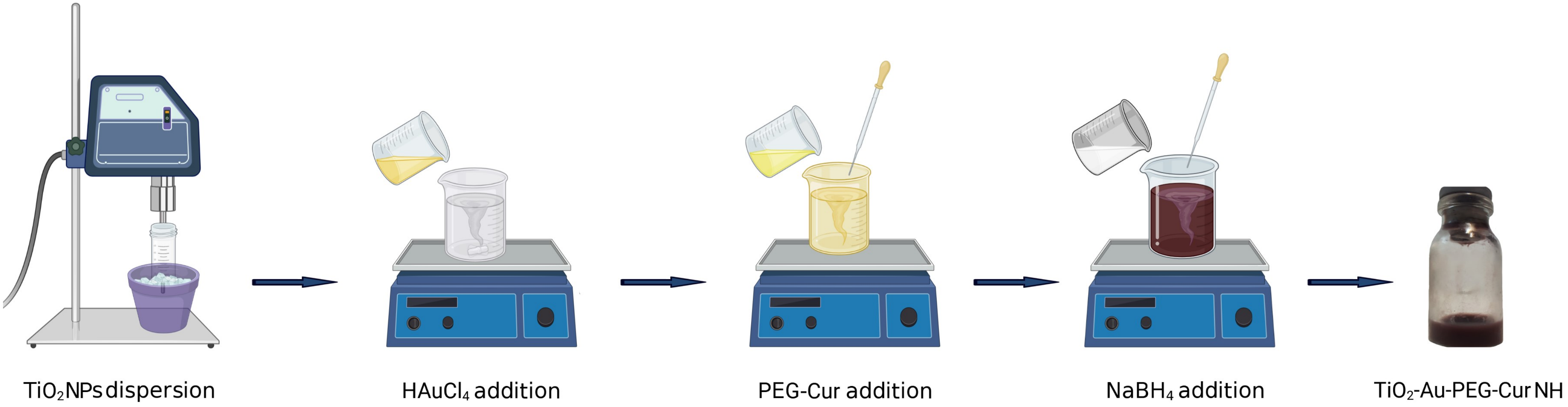

TiO_2 NPs dispersion
$HAuCl_4$ addition
PEG-Cur addition
$NaBH_4$ addition
TiO_2-Au-PEG-Cur NH
