# Supplementary figures and images for: An amplified sonodynamic therapy by a nanohybrid of titanium dioxide-gold-polyethylene glycol-curcumin: HeLa cancer cells treatment in 2D monolayer and 3D spheroid models
Source: Ultrason Sonochem. 2023 Dec 25;102:106747. doi: 10.1016/j.ultsonch.2023.106747 (PMC10765485; doi:10.1016/j.ultsonch.2023.106747)

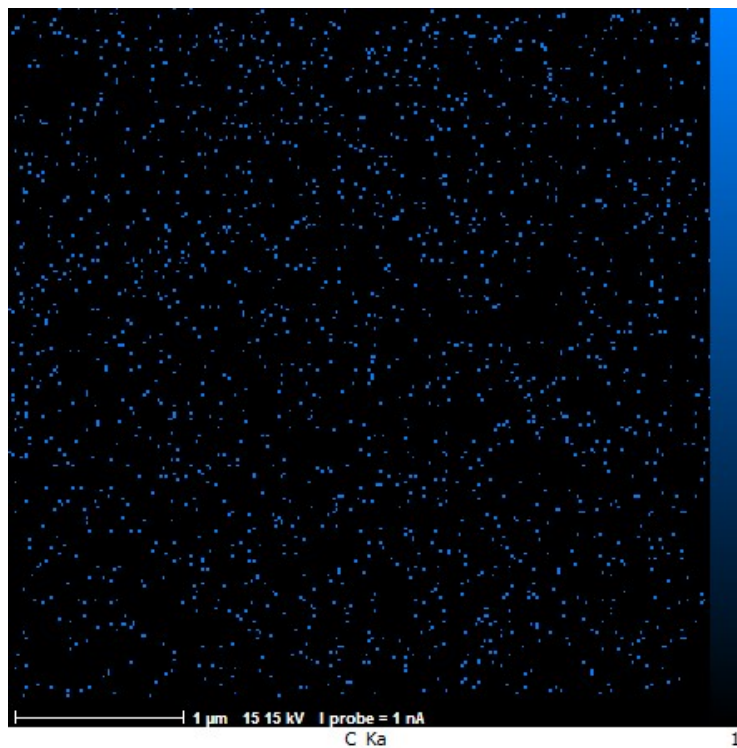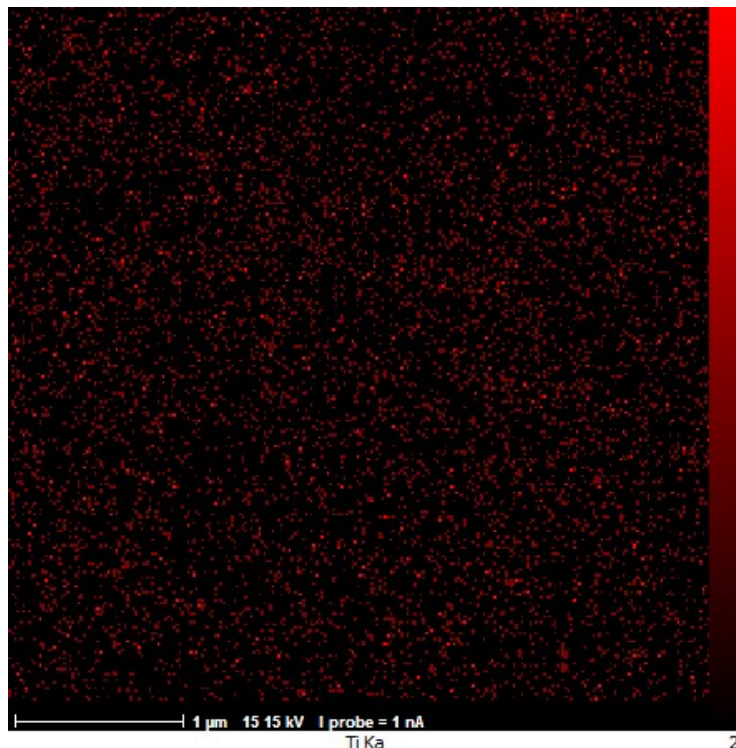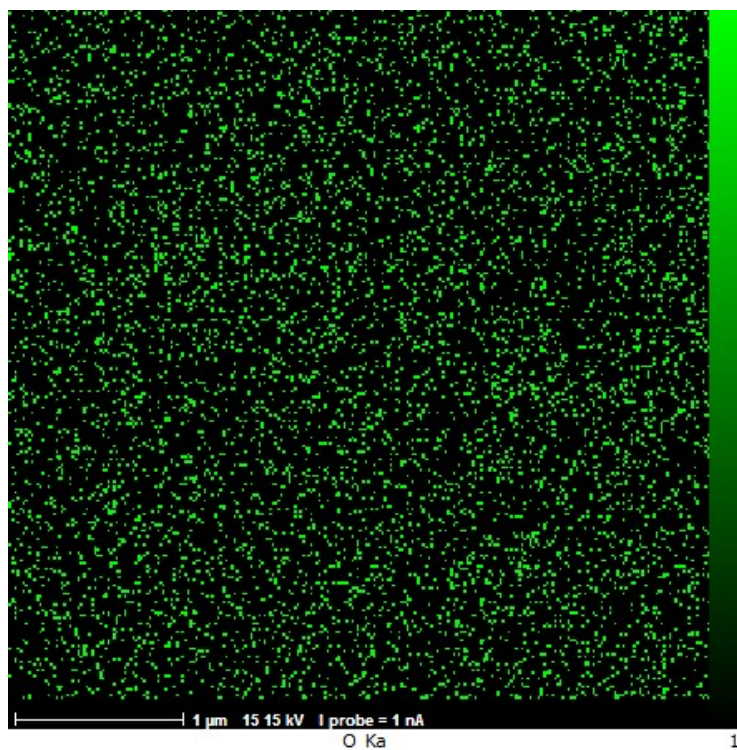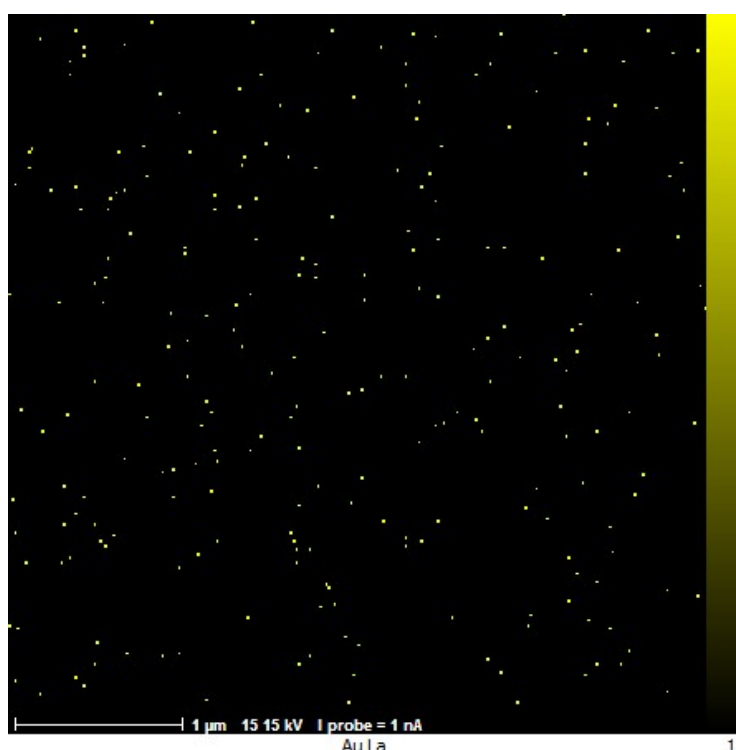

Supplement: Supplementary data 3 [file mmc3.pdf]

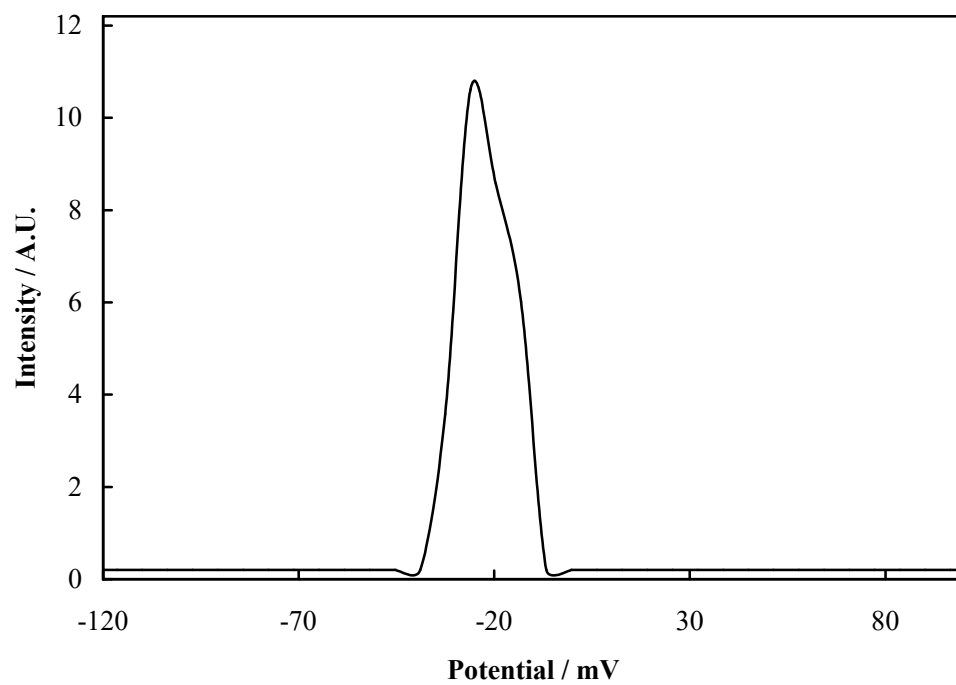

Supplement: Supplementary data 4 [file mmc4.pdf]

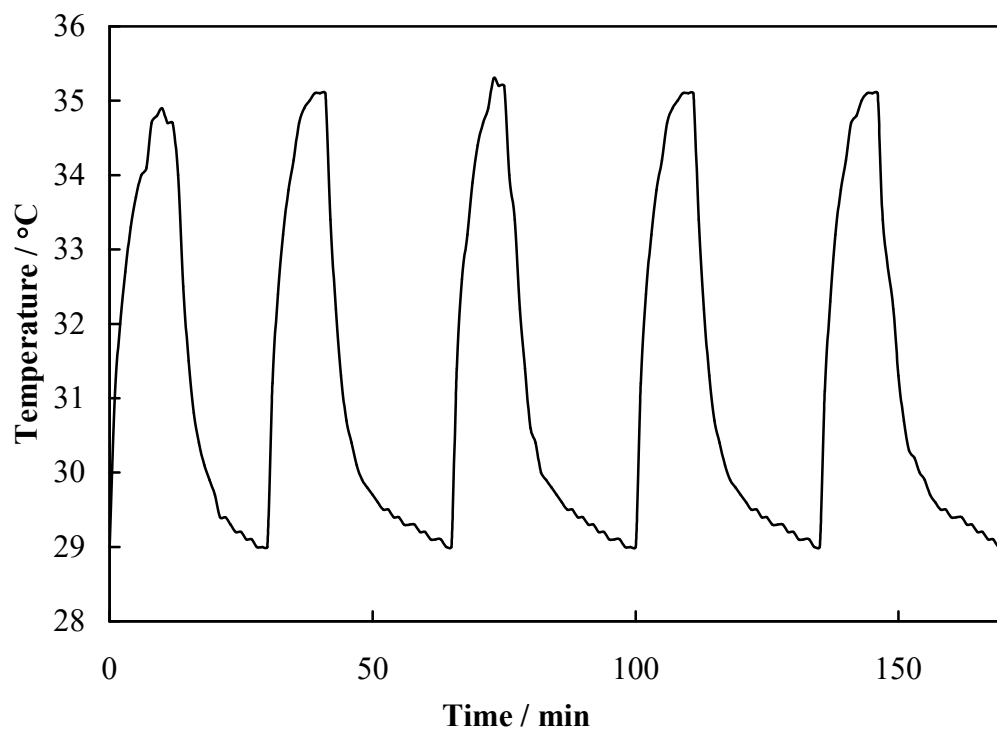

Supplement: Supplementary data 5 [file mmc5.pdf]

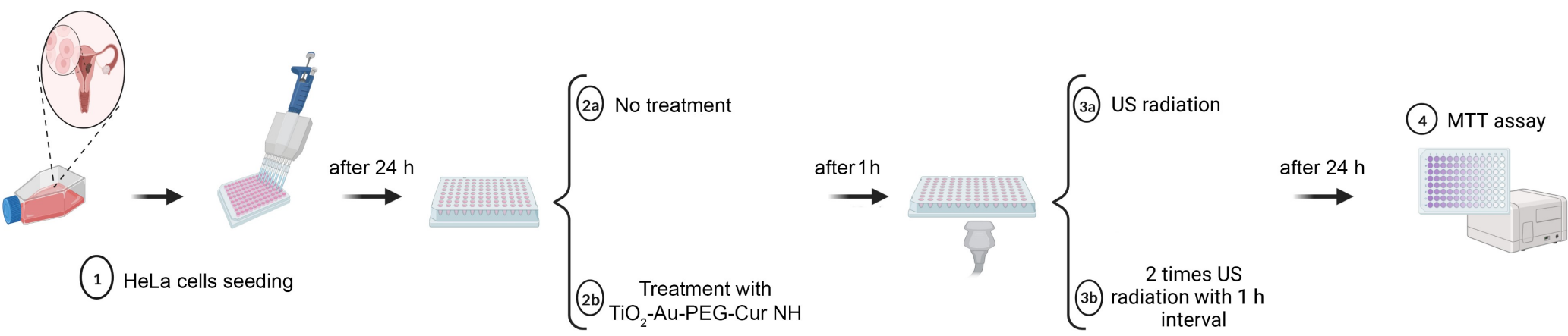

Supplement: Supplementary data 6 [file mmc6.pdf]

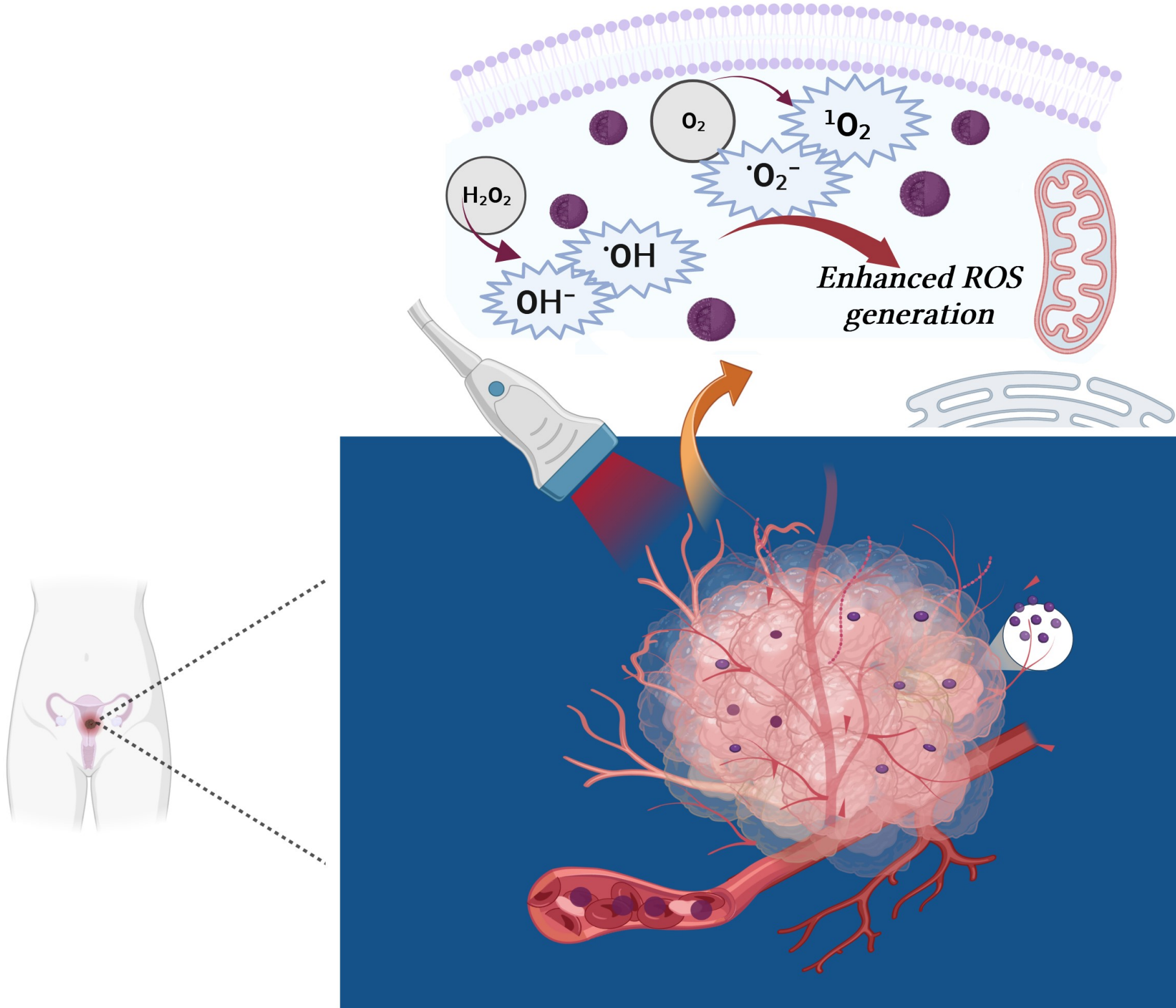

Supplement: Supplementary data 9 [file mmc9.pdf]

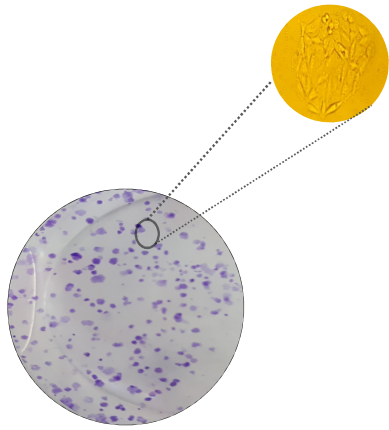

**US-/NH-**

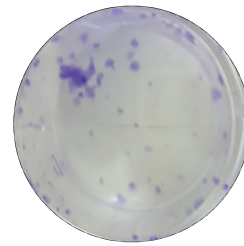

**US+1/NH-**

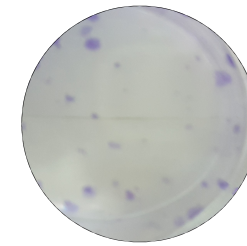

**US+2/NH-**

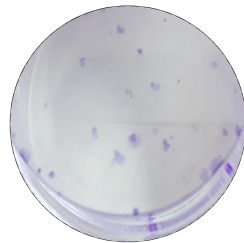

**US-/NH+**

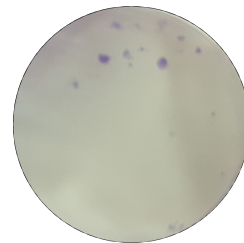

**US+1/NH+**

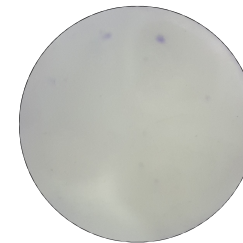

**US+2/NH+**

Supplement: Supplementary data 10 [file mmc10.pdf]

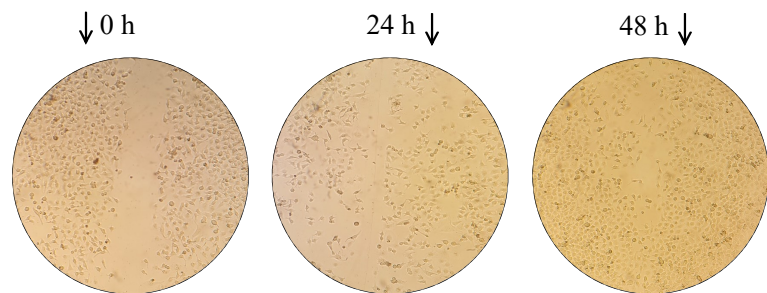

**US-/NH+**

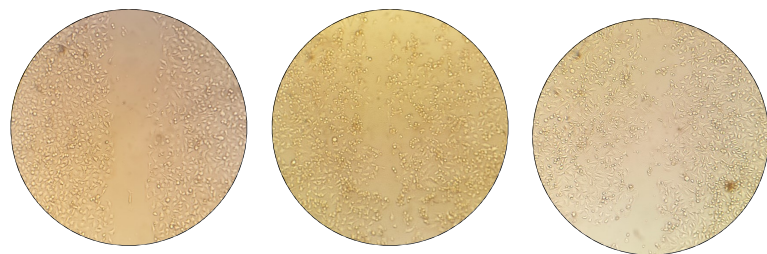

**US+2/NH-**

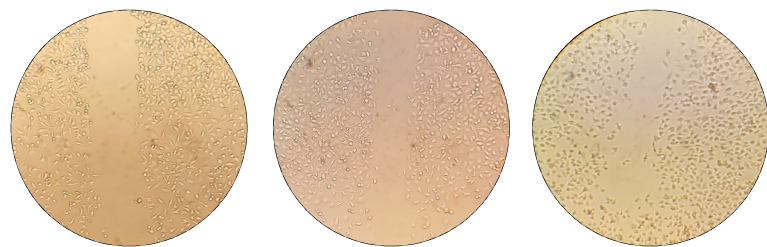

**US+2/NH+**

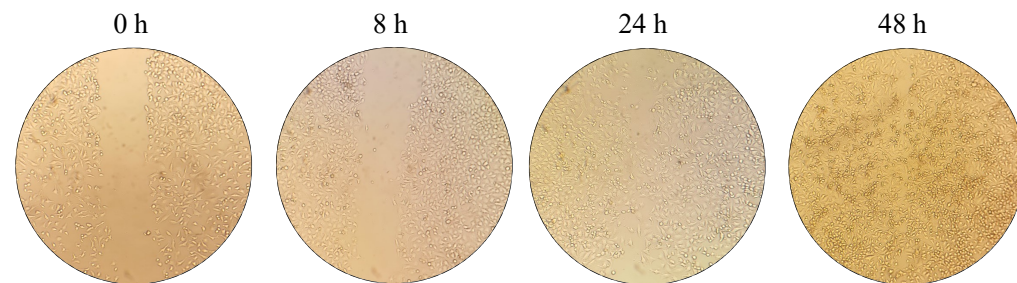

**US-/NH-**

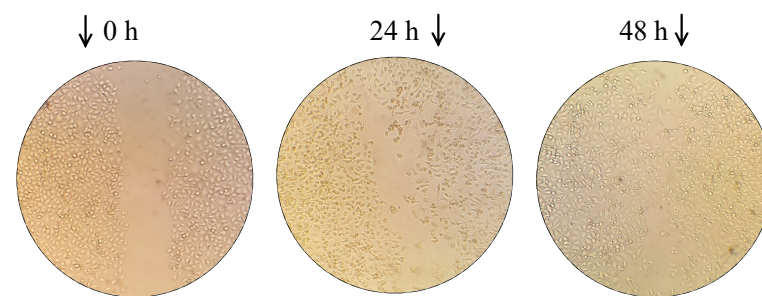

**US+1/NH-**

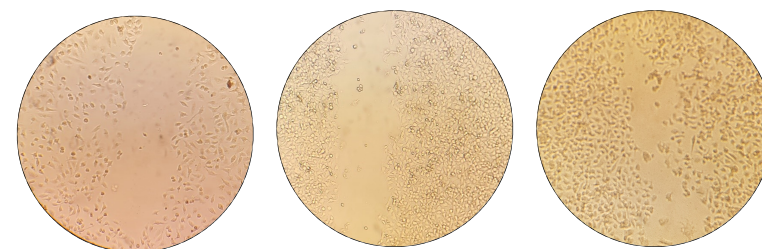

**US+1/NH+**

Supplement: Supplementary data 11 [file mmc11.pdf]

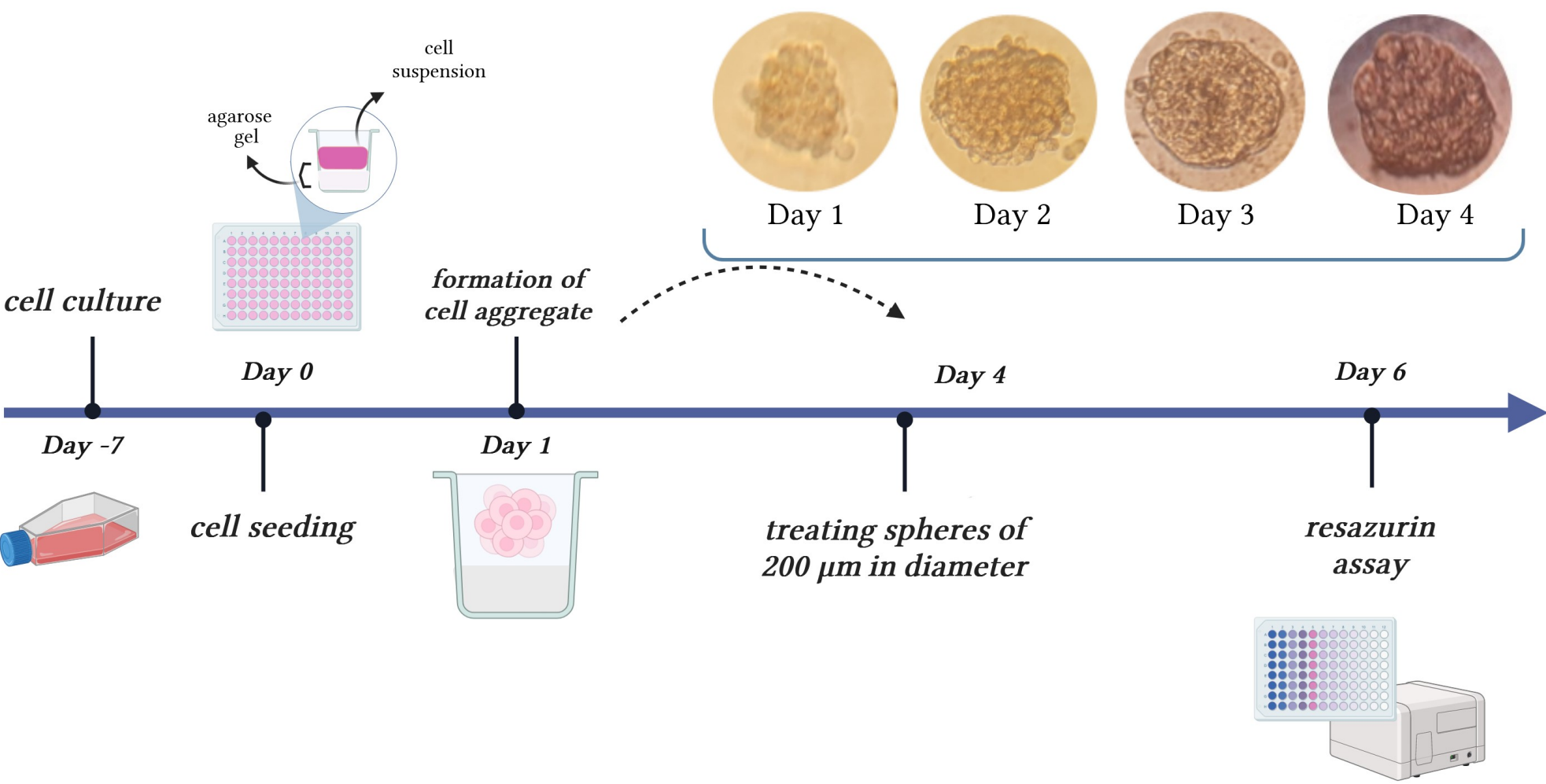

Supplement: Supplementary data 12 [file mmc12.pdf]
