# Supplementary material for: An amplified sonodynamic therapy by a nanohybrid of titanium dioxide-gold-polyethylene glycol-curcumin: HeLa cancer cells treatment in 2D monolayer and 3D spheroid models
Source: Ultrason Sonochem. 2023 Dec 25;102:106747. doi: 10.1016/j.ultsonch.2023.106747 (PMC10765485; doi:10.1016/j.ultsonch.2023.106747)
Supplement: Supplementary data 7 [file mmc7.pdf]

Type of NPs	IC50 / µg mL^{-1}	Type of cells	Ref.
TiO_2	5408.8±45.9	U-2 OS (osteosarcoma)	[1]
TiO_2	211.3±15.2	SW 1353 (chondrosarcoma)	[1]
TiO_2	74.29±8.95	HeLa	[2]
TiO_2	82.02±6.03	DU-145 (Prostate)	[2]
TiO_2 impregnated with Pt	53.74±2.95	HeLa	[2]
TiO_2	43.85	HeLa	[3]
TiO_2	35.09	HEK293 (kidney)	[3]
CuO	22.48	HeLa	[3]
TiO_2	29.73	HeLa	[4]
TiO_2+Al_2O_3	4.07±0.81	HeLa	[5]
TiO_2	16.63±1.41	HeLa	[5]
Catharanthus roseus Au	5	HeLa	[6]
Au	152.16	HeLa	[7]
Au	196.32	K562 (Myeloid Leukemia)	[7]
Au	104	HeLa	[7]

References:

[1] Jubu, P.R., et al., Dispensability of the conventional Tauc's plot for accurate bandgap determination from UV–vis optical diffuse reflectance data. Results in Optics, 2022. 9: p. 100273.

[2] González-Larraza, P.G., et al., IC(50) Evaluation of Platinum Nanocatalysts for Cancer Treatment in Fibroblast, HeLa, and DU-145 Cell Lines. ACS Omega, 2020. 5(39): p. 25381-25389.

[3] Gamedze, N.P., et al., Physico-chemical characteristics and cytotoxicity evaluation of CuO and TiO2 nanoparticles biosynthesized using extracts of Mucuna pruriens utilis seeds. Heliyon, 2022. 8(8): p. e10187.

[4] Mbenga, Y., et al., Green synthesis, antioxidant and anticancer activities of TiO2 nanoparticles using aqueous extract of Tulbhagia violacea. Results in Chemistry, 2023. 6: p. 101007.

[5] Cakmak, N., A. Taş, and Y. Silig, Evaluation of Synergistic Effect of TiO2 and Al2O3 Nanoparticles on Hela Cell Line. 2018. 4: p. 2018.

[6] Ke, Y., et al., Photosynthesized gold nanoparticles from Catharanthus roseus induces caspase-mediated apoptosis in cervical cancer cells (HeLa). Artif Cells Nanomed Biotechnol, 2019. 47(1): p. 1938-1946.

[7] Aljarba, N.H., et al., Anticancer and microbial activities of gold nanoparticles: A mechanistic review. Journal of King Saud University - Science, 2022. 34(4): p. 101907.
