# Supplementary material for: An amplified sonodynamic therapy by a nanohybrid of titanium dioxide-gold-polyethylene glycol-curcumin: HeLa cancer cells treatment in 2D monolayer and 3D spheroid models
Source: Ultrason Sonochem. 2023 Dec 25;102:106747. doi: 10.1016/j.ultsonch.2023.106747 (PMC10765485; doi:10.1016/j.ultsonch.2023.106747)
Supplement: Supplementary data 8 [file mmc8.pdf]

Ref.	Sonosensitizer	Cell line	Concentration / µg mL^{-1}	Cytotoxicity without US radiation	Cytotoxicity with US radiation	Ultrasound Parameters			
						Frequency / MHz	Power / W cm^{-2}	Duration / s	Duty cycle
[1]	Alginate-coated Au nanorods	MDA-MB-231	200	-	31.2% (24h)	1	1	180	50%
[2]	Au-TiO_2	MCF7	100	-	57.6% (24h)	1	1.5	300	-
[3]	Cur-Au NPs-PEG	B16/F10	25	70% (24h)	<10% (24h)	1	1	60	100%
[4]	Au NPs	HeLa	5	94% (24h)	75% (24h)	1	1	60	
[5]	Cur-Au NCs	HeLa	100	48% (24h)	-	-	-	-	-
[6]	TiO_2	Melanoma	0.05%	92.4%	53.6%	1	1	10	50%
[7]	Au/TiO_2 NCs	B16/F10	100	74% (72h)	20% (72h)	1	1	60	100%
[8]	Au/TiO_2 NPs	MCF7	60	52% (24h)	-	-	-	-	-
[9]	Au doped TiO_2	MCF7	400	21% (24h)	-	-	-	-	-
This study	TiO_2-Au-PEG-Cur NH	HeLa	100	54% (24h)	43% (24h)	1.0	1.0	600	10%

References:

[1] Y.L. Loke, A. Beishenaliev, P.W. Wang, C.Y. Lin, C.Y. Chang, Y.Y. Foo, F.N. Faruqu, B.F. Leo, M. Misran, L.Y. Chung, D.B. Shieh, L.V. Kiew, C.C. Chang, Y.Y. Teo, ROS-generating alginate-coated gold nanorods as biocompatible nanosonosensitisers for effective sonodynamic therapy of cancer, Ultrasonics sonochemistry 96 (2023) 106437. https://doi.org/10.1016/j.ultsonch.2023.106437

[2] Cao, Yu & Wu, Tingting & Dai, Wenhao & Dong, Haifeng & Zhang, Xueji, TiO2 Nanosheets with Au Nanocrystals Decorated Edge for Mitochondria-Targeting Enhanced Sonodynamic Therapy, Chemistry of Materials (2019). 2019. 10.1021/acs.chemmater.9b03430.

[3] Z. Kayani, R. Dehdari Vais, E. Soratijahromi, S. Mohammadi, N. Sattarahmady, Curcumin-gold-polyethylene glycol nanoparticles as a nanosensitizer for photothermal and sonodynamic therapies: In vitro and animal model studies, Photodiagnosis and photodynamic therapy 33 (2021) 102139. https://doi.org/10.1016/j.pdpdt.2020.102139

[4] A. Shanei, H. Akbari-Zadeh, Investigating the Sonodynamic-Radiosensitivity Effect of Gold Nanoparticles on HeLa Cervical Cancer Cells, Journal of Korean medical science 34(37) (2019) e243. https://doi.org/10.3346/jkms.2019.34.e243
[5] S. Govindaraju, A. Rengaraj, R. Arivazhagan, Y.S. Huh, K. Yun, Curcumin-Conjugated Gold Clusters for Bioimaging and Anticancer Applications, Bioconjugate chemistry 29(2) (2018)363–370. https://doi.org/10.1021/acs.bioconjchem.7b00683
[6] Y. Harada, K. Ogawa, Y. Irie, H. Endo, L.B. Feril, T. Jr, Uemura, K. Tachibana, Ultrasound activation of TiO2 in melanoma tumors, Journal of controlled release : official journal of the Controlled Release Society 149(2) (2011) 190–195. https://doi.org/10.1016/j.jconrel.2010.10.012
[7] G. Perota, G. N. Zahraie, R. Dehdari Vais, M.H. Zare, N. Sattarahmady, Au/TiO2 nanocomposite as a triple-sensitizer for 808 and 650 nm phototherapy and sonotherapy: Synergistic therapy of melanoma cancer in vitro, Journal of Drug Delivery Science and Technology 76 (2022) 103787. 10.1016/j.jddst.2022.103787.
[8] M. Akram, M. Fakhar-e-Alam, M. Aziz, Dr. Muhammad & Alimgeer, Khurram & Atif, M. & Amir, M. & Hanif, Atif & Farooq, Aslam, Tailoring of Au-TiO2 Nanoparticles conjugated with Doxorubicin for their Synergistic Response and Photodynamic Therapy Applications, Journal of Photochemistry and Photobiology A: Chemistry 384 (2019) 112040. 10.1016/j.jphotochem.2019.112040.
[9] S. Iqbal, M. Fakhar-E-Alam, K.S. Alimgeer, M. Atif, A. Hanif, N. Yaqub, W.A. Farooq, S. Ahmad, Y.M. Chu, M. Suleman Rana, A. Fatehmulla, H. Ahmad, Mathematical modeling and experimental analysis of the efficacy of photodynamic therapy in conjunction with photo thermal therapy and PEG-coated Au-doped TiO2 nanostructures to target MCF-7 cancerous cells, Saudi journal of biological sciences 28(2) (2021) 1226–1232. https://doi.org/10.1016/j.sjbs.2020.11.086
